# Anomalous shape effect of nanosized helium bubble on the elastic field in irradiated tungsten

**DOI:** 10.1038/s41598-020-80167-7

**Published:** 2021-01-12

**Authors:** Xinlong Huang, Chenyangtao Lv, Haijian Chu

**Affiliations:** 1 School of Mechanics and Engineering Science, Shanghai University, Shanghai, 200444 China; 2 Shanghai Institute of Applied Mathematics and Mechanics, Shanghai University, Shanghai, 200444 China; 3 Shanghai Key Laboratory of Energy Engineering Mechanics, Shanghai University, Shanghai, 200444 China

**Keywords:** Nanoscale materials, Structural properties, Structural materials

## Abstract

Bubble pressure and elastic response in helium-irradiated tungsten are systematically investigated in this study. An anomalous shape effect is found that the radial normal stress and mean stress distributions around a nanosized void or bubble are far from the spherical symmetry, which is ascribed to polyhedral geometry characteristic of the nanosized bubble and physical mechanism transition from crystal surfaces dominated to the surface ledges and triple junctions dominated. Molecular simulation shows that Young–Laplace equation is not suitable for directly predicting equilibrium pressure for nanosized bubble in crystals. Consequently, a new criterion of average radial normal stress of spherical shell is proposed to polish the concept of equilibrium pressure of helium bubbles. Moreover, the dependences of bubble size, temperature and helium/vacancy ratio (He/Vac ratio) on the bubble pressure are all documented, which may provide an insight into the understanding of mechanical properties of helium-irradiated tungsten.

## Introduction

Structural materials used in nuclear reactors are required to tolerate extreme operating conditions such as high temperature, high stress, intense radiation fluxes and chemical erosion, etc^1^. He atoms are introduced into metals mainly ascribed to deuterium–tritium fusion^2^ and (n, α) reactions^3^ and tend to aggregate into bubbles at lattice defect sites such as vacancy clusters and grain boundaries^4^. In fusion reactors, the bombardment of He atoms and high-energy neutrons may result in the damage and the formation of helium bubble^5^. As a good radiation resistant material, tungsten has been applied in both in fission and fusion reactors. Particularly, tungsten is regarded as the primary candidate to face the plasma bombardment in the diverter in ITER^6^. During the service of these reactors, the pressure of the helium bubble inside tungsten or some other radiation resistant materials may increase due to the successive He generation or implantation. The over-pressurized He bubbles grow through loop punching mechanism by emitting prismatic dislocation loops^7^ or even rupture on the tungsten surface which may result in “fuzz” nanostructure^8^. Therefore, the influence of He bubble on the mechanical properties of irradiated tungsten is significant to the safety of nuclear energy engineering.

Bubble size and its density are the two key parameters to characterize the microstructure of the helium bubbles in tungsten, which is dependent on the irradiation environment or conditions including temperature, incident helium ion energy, flux and fluence. Sikka et al.^9^ calculated the average size and density of the voids in fast-neutron-irradiated W at 823 K with a fluence of approximately 10^22^ neutrons cm^−2^. The void size is at the range of 1–6 nm in diameter, while the average value is about 3 nm. Harrison et al.^5^ found that the He bubble is 3.1 ± 0.4 nm in tungsten irradiated with 15 keV He ions at 773 K and with the dose of 3.0 dpa. Nishijima et al.^10^ observed that the bubble size increases dramatically when the temperature is higher than the recrystallization temperature of W (1400–1500 K), while the diameter of helium bubbles is less than 5 nm at 1300 K. It is also reported that the He bubble size increases with temperature^11^, whereas its density decreases with the increase of temperature^12^.

The He/Vac ratio and bubble pressure in metals affect the mechanical properties of materials to a great extent, which may cause hardening and strain localization in matrix^13^. Based on the assumption that the volume expansion of irradiated sample is totally ascribed to implanted He, Jager et al.^14^ found that the He/Vac ratio is 1.1 in Ni at room temperature by step height measurement. Li et al.^15^ observed that the He/Vac ratio is 0.83 in Cu at room temperature by Transmission Electron Microscopy (TEM). Recently, the combination of in-situ Scanning Transmission Electron Microscopes (STEM) and Electron Energy Loss Spectroscopy (EELS) are developed to measure He/Vac ratio. For instance, David et al.^16^ applied this method and reported that He/Vac ratio is 2.13 in Si at 300 K. It should be noted that this method requires complex theoretical formulas and is restricted to the sample containing small helium bubbles^17^. So far, since no experimental method is available to obtain the bubble pressure, two indirect methods, i.e., molecular simulation (MS) and theoretical prediction are often applied. By MS, Ito et al.^18^ studied the internal pressure of a 2 nm helium bubble in tungsten with different He/Vac ratios at the range of 1–7, and found that the pressure is at the range of 1–500GPa. Comparing the pressure in the bubble in tungsten and that in pure helium under the condition of same He/Vac ratio or helium density, Cui et al.^19^ reported that He/Vac ratio is the dominated factor to the bubble pressure and the influence of matrix can be neglected. Based on the balance of internal pressure and surface tension, Young–Laplace equation is often used to predict the equilibrium pressure which is a basic parameter for the irradiated material. For instance, by this equation Ding et al.^20^ estimated the equilibrium pressure in helium bubble with average diameter of 6.6 nm is ~ 1.0GPa in Cu. Sefta et al.^21^ used Young–Laplace equation to estimate the equilibrium pressure is 3.03GPa for the 3.8 nm He bubble in tungsten and got the He/Vac ratio is 0.9 by state equation of helium. However, the reliability of the Young–Laplace equation for bubbles at the nanoscale is somewhat questionable due to the fact that it is originally deduced based on the ideal configuration of bubbles in the framework of macroscale continuum mechanics. Actually, the He bubble is surrounded by faceted tungsten crystal planes. The influence of the non-spherical shape of the nanosized bubble on its equilibrium pressure and the elastic response of the surrounding matrix or tungsten have not been investigated systematically.

In this article, the effects of bubble shape, bubble size, temperature and the He/Vac ratio on the bubble pressure and the elastic response in tungsten are investigated. An anomalous shape effect of nanosized voids and helium bubbles is reported, and its corresponding mechanism is uncovered from the aspects of geometry and physics. The concept of equilibrium pressure is polished and a criterion of average radial normal stress of spherical shells is proposed to get the equilibrium pressure.

## Model and methodology

**Figure 1:**
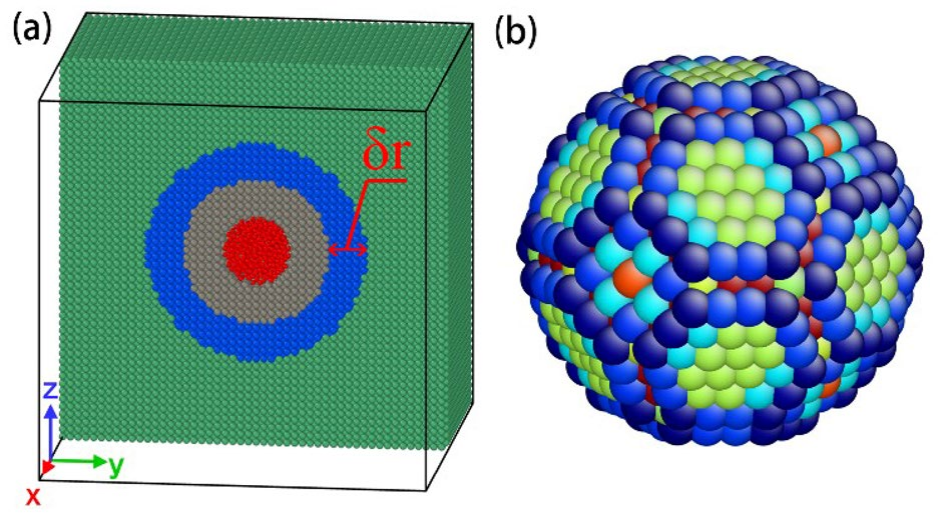
A helium bubble-embedded tungsten model (**a**) and the configuration of the nearest tungsten atoms surrounding the bubble (**b**). A spherical shell with thickness of $\mathrm{\delta r}$ is marked in dashed red circle, which is set to evaluate the average stress.

A model of spherical nanosized bubble-embedded tungsten is created as shown in Fig. 1a. The dimensions are 50*a* × 50*a* × 50*a* where *a* is the lattice constant of tungsten. The axes X, Y and Z are along [100], [010] and [001], respectively. Periodical boundary conditions are applied during the molecular simulation. The Nose–Hoover method is applied to control the system temperature and the simulation time step is set to 1 fs. Models are relaxed for 160 ps under NVT ensemble. Generally, the total energy of the system with inherent temperature-induced fluctuation will be convergent in 10 ps relaxation. The polyhedron in Fig. 1b shows the nearest tungsten atoms surrounding the bubble, which indicates that the configuration of the bubble is not exactly spherical.

The process to create the spherical bubble is as follows: (1) Create a pure tungsten model based on its BCC lattices and relax to no-stress state; (2) Delete the atoms in the spherical region with specified radius surrounding the central atom in the model and get a spherical void inside the model (for instance, 893 atoms are deleted for the case of 3 nm in diameter); (3) Insert helium atoms into the void and get the bubble-embedded tungsten model; (4) Relax the model under the concerned temperature. In this article, the bubble size is set to 2–10 nm. The original model of pure tungsten includes 250,000 atoms for the cases of 2–7 nm, while the atom number increases to 2,000,000 for the cases of 8 nm and 10 nm in order to reduce the influence of model boundary. The practical atom number of bubble-embedded model varies according to the bubble size and the He/Vac ratio. In order to evaluate bubble-induced radial normal stress in tungsten, a spherical shell region (the blue shell region as shown in Fig. 1a) surrounding the bubble is applied.

The potential applied in this article is referred to the work of Juslin and Wirth where the N-body potential for tungsten was originally developed by Finnis and Sinclair^22^, and subsequently modified by Ackland and Thetford^23^ and Juslin and Wirth^24^. The W-He potential was developed by Juslin and Wirth^24^. The interatomic potential for helium was obtained from Beck^25,26^. The reliability of this potential is well validated according to Sandoval et al.^27^ and Hammond et al.^28^ The software Lammps is applied for the MS. Both Atomeye^29^ and Ovito^30^ are used to visualize atoms.

The surface energy of the nanovoid can cause the tungsten atoms at the helium-tungsten interface to be subjected to radial normal stress pointing to the center of the sphere. Since the atom displacements and corresponding stress fields in the interface layer are relatively rough, we set a spherical shell with a thickness of 1.5 nm (the gray area in Fig. 1a) in order to exclude the influence of the interface layer. In other word, the spherical shell region (the blue region in Fig. 1a) with a thickness δr of 1–3 nm is used as the monitored region to evaluate the effect of helium bubbles on tungsten matrix, and the average radial normal stress of the monitored shells is calculated, i.e.

1 $$\overline{{\sigma_{rr} }} = \frac{1}{V}\int_{V} {\sigma_{rr} {\text{d}}V} = \frac{1}{N}\sum\limits_{i = 1}^{N} {\sigma_{rr} }$$

where *N* is the atom number in the concerned shell occupied by volume *V*. $\sigma_{rr}$ is the radial normal stress.

The Young–Laplace equation are often applied to estimate the influence of surface energy, i.e.

2 $$p = {4}\gamma /d$$

where *p* is the equivalent internal pressure to balance the surface tension, named equilibrium pressure, γ is the surface energy and *d* the diameter of the bubble. In the following, the average radial normal stress will be calculated based on the results of molecular simulation and the reliability of Young–Laplace equation will be evaluated. Since bubbles are surrounded by a few facets of tungsten and different facets or crystal planes often have different surface energy, four crystal planes (100), (110), (111) and (112) with low Miller indices have been simulated to get the surface energy and their corresponding results are 3.291 J m^−2^, 3.409 J m^−2^, 3.268 J m^−2^, 3.227 J m^−2^ at 300 K, respectively. The surface energy of crystal plane is obtained by routine simulation of the typical “slab” approach^31^. Details are not given here for brevity. Their average 3.299 J m^−2^ is consistent with the experiment result (3.265 J m^−2^)^32^, which will be applied in Eq. (2) to get the equivalent equilibrium pressure in this work.

## Results

### Shape effect of the nanovoid on the elastic field

**Figure 2:**
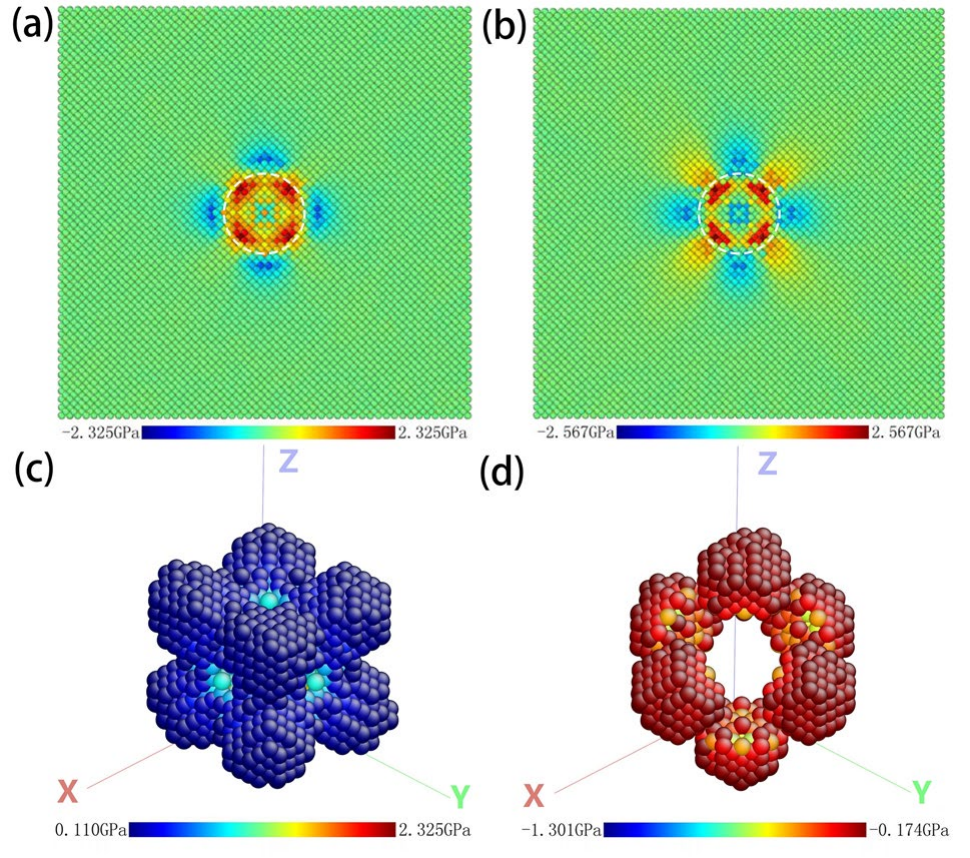
Stress distribution of the void-embedded tungsten model where the void diameter is 3 nm: (a) the mean stress contour from the projection along [100]; (**b**) radial normal stress contour from the projection along [100]; (**c**) configuration of the expansion region surrounding the nanovoid; (**d**) configuration of the contraction region surrounding the nanovoid. The simulation temperature is 300 K and no helium atoms are in the void. White dotted circles in (**a**,**b**) represent the outline of the nanovoid.

The stress distribution in the model of tungsten embedded a void with 3 nm in diameter is analysed and given in Fig. 2 where the mean stress is

3 $$\sigma_{{\text{m}}} = \tfrac{1}{3}\left( {\sigma_{xx} + \sigma_{yy} + \sigma_{zz} } \right)$$

According to the linear elasticity, the mean stress is proportional to the volume strain. Thus positive mean stress implies the volume expansion while the negative the contraction. In order to reduce the temperature-induced fluctuation, the stress at each point (atom) is obtained by the time average in 40 ps and by spatial average within adjacent spherical region with 0.5 nm radius.

From Fig. 2a,b, we can find that both the mean stress and radial normal stress are not spherical symmetrical and their values vary with the directions. By recalling the prediction of Young–Laplace equation, the surface tension of the void would lead to radial tensile stress in the matrix, and both the normal stress and radial stress should be spherically symmetric. The reason for this anomalous phenomenon is ascribed to the shape of the nanosized void. Actually, from the geometric aspect, the nanosized void is polyhedral as shown in Fig. 1b, which is far from the smooth spherical. Since the smooth condition is fundamental for the derivation of Young–Laplace equation in Eq. (2), the empirical formula to compute the equilibrium pressure is not strict or even invalid for nanovoid in crystals. From the aspect of energy, as the void size decreases, the contributions of ledges and triple intersection junctions of crystal planes (as shown in Fig. 1b) to the surface energy gradually increase and become dominated. Therefore, the mechanism of surface energy induced stress field dominated by the crystal planes would convert to that by the ledges and triple intersection junctions. Figure 2c,d show the configurations of the expansion regions with positive mean stress and the contraction regions with negative mean stress surrounding the nanovoid, respectively. It can be found that there exist 8 expansion regions in Fig. 2c and 6 contraction ones in Fig. 2d. The stress field in Fig. 2 show elegant axial symmetry (not spherical symmetry), which is ascribed to the crystal symmetry of BCC tungsten and geometry symmetry of the void. In addition, overall, the magnitude of the stress is gradually decreased with the increase of the distance from the void. It should be noted that the anomalous distribution of stress field is not attributed to the anisotropy of materials, since the concerned tungsten is a well-known most-likely isotropic material.

### The size effect on equilibrium pressure

Surface energy of the nanosized helium bubble and its internal pressure are two key factors to the understanding of the mechanical behavior of helium irradiated metallic materials. Overall, the former would lead to the shrink of the bubble and the surrounding matrix is stretched, whereas the latter will cause helium bubbles to expand and exert pressure on the matrix. Therefore, one may expect there exists a critical or equilibrium state where the two effects can neutralize each other. However, because of the abnormal shape effect found in the last section, the stress field caused by nanovoids or helium bubbles cannot be eliminated completely. Therefore, we propose a criterion to predict the equilibrium pressure of the helium bubble. In other words, if the average radial normal stress of tungsten matrix in the monitored spherical shells around the helium bubble is zero, then the internal pressure of the helium bubble is the equilibrium pressure and the corresponding He/Vac ratio is called equilibrium He/Vac ratio. This criterion means that under the action of surface energy and equilibrium bubble pressure, the overall radial normal stress of the monitored spherical shells is zero.

**Figure 3:**
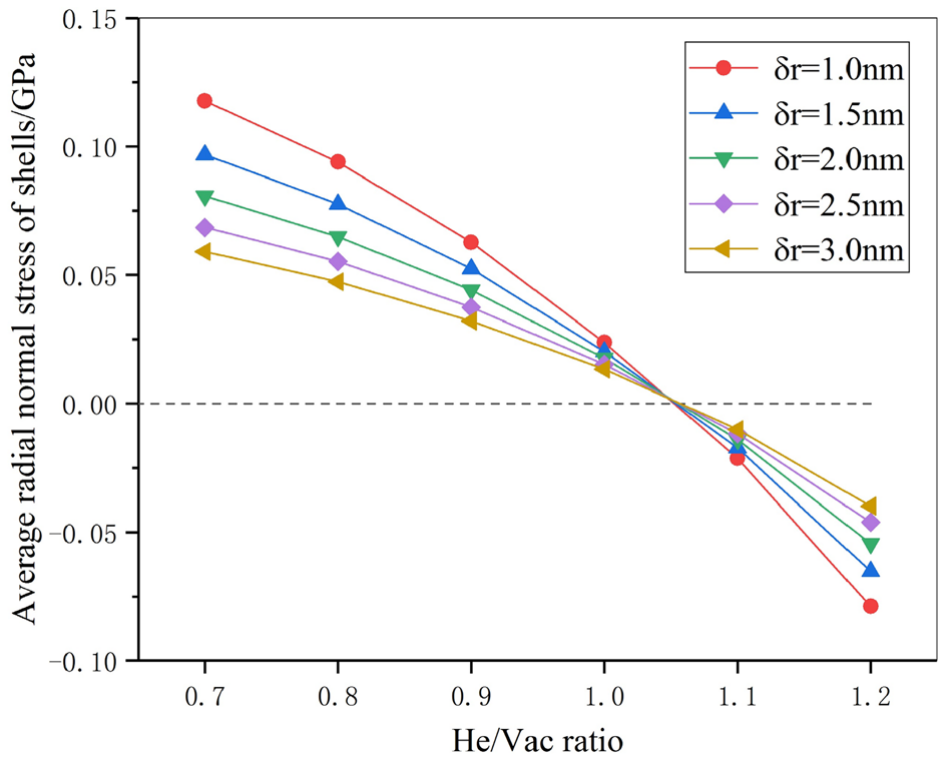
The average radial normal stress of tungsten in monitored shells with respect to He/Vac ratio regarding different shell thickness.

Figure 3 shows the average radial normal stress in the monitored spherical shells with respect to He/Vac ratio where five different thicknesses δ*r* (as shown in Fig. 1a) are considered. During the simulation, NVT ensemble is adopted and the temperature is set to 300 K. The helium bubble diameter is 3 nm. We can find that: (1) when the He/Vac ratio is small, the average radial normal stresses of the tungsten matrix in the spherical shells are all positive, indicating that the spherical shells are under tension and the stress is dominated by the surface energy. (2) With the increase of He/Vac ratio, the average radial normal stress decreases from positive to negative, which corresponds to the transition from surface energy dominated to bubble pressure dominated. (3) There is a critical state in which the average radial normal stress of tungsten matrix in spherical shells is zero. Different from other states, the average radial normal stress at the critical state is not sensitive to the shell thickness, and all equal zero. Therefore, it can be deduced that the overall effects due to the surface energy and bubble pressure are neutralized regarding the average radial normal stress. The He/Vac ratio at this state is the equilibrium ratio, which equals to 1.05 by the method of interpolation. The corresponding equilibrium pressure is 2.08 GPa.

**Figure 4:**
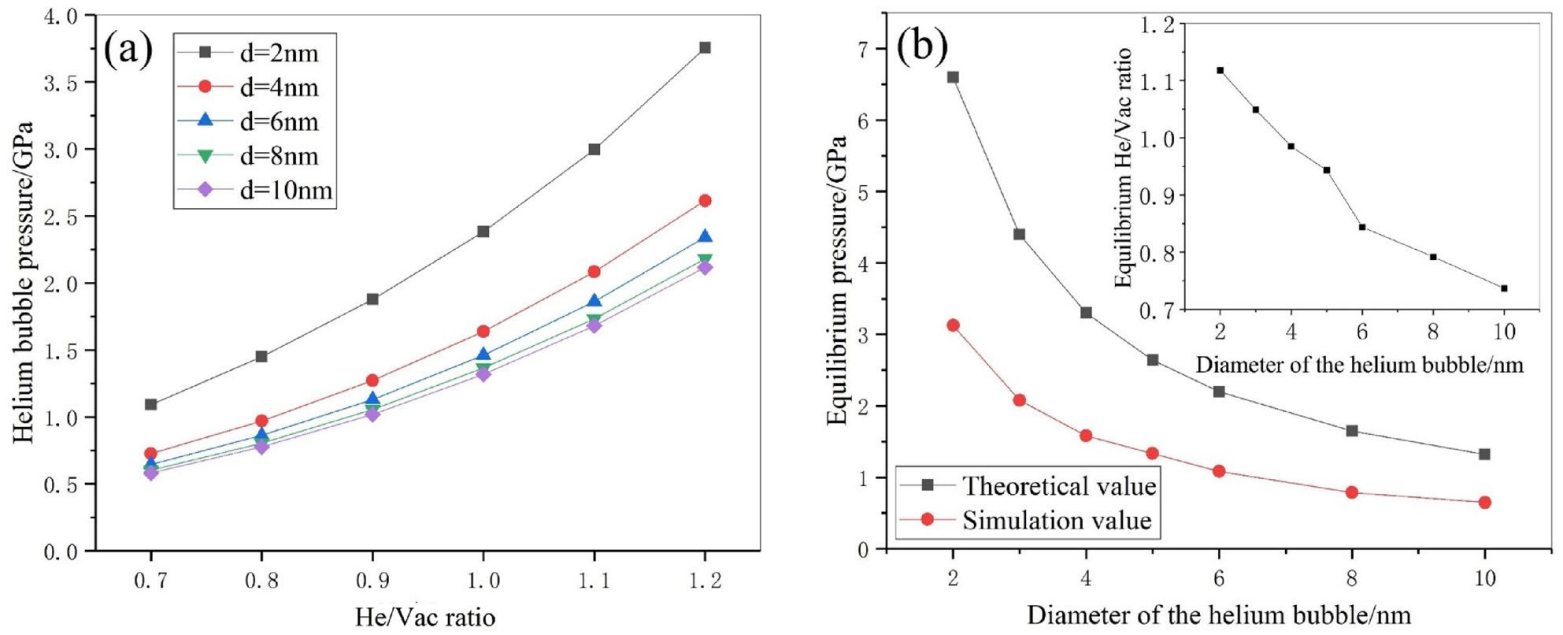
(**a**) Helium bubble pressure with respect to He/Vac ratio regarding different bubble sizes. (**b**) Equilibrium pressure and equilibrium He/Vac ratio with respect to the size of helium bubble.

By the similar method, we can obtain the critical state for the helium bubble with different sizes and results are shown in Fig. 4. Figure 4a gives the dependence of bubble pressure with respect to He/Vac ratio. As expected, the pressure increases with the He/Vac ratio. With the increase of the bubble size, the size effect on the pressure decreases. The pressure for *d* = 8 nm is very close to that for *d* = 10 nm as shown in Fig. 4a. It is noted that the obtained pressure for the case of 10 nm coincides with the experiment results for the pure hellium^33^. Figure 4b shows the size dependence of equilibrium pressure, in which the theoretical prediction by Eq. (2) is given for comparison. We can find that: (1) both molecular simulation and theoretical prediction shows that the equilibrium pressure increases with the decrease of the bubble size. (2) The equilibrium pressure obtained by molecular simulation is less than theoretical prediction. For example, by molecular simulation, the equilibrium pressure of a helium bubble with 3 nm in diameter is 2.08 GPa, whereas, by Young–Laplace equation, it is 4.40 GPa. The distinct difference between the simulation and theory implies that the ideal theoretical formula overestimates the equilibrium pressure for helium bubbles in tungsten and may be not suitable for nanoscale crystal materials.

### Temperature dependence on equilibrium pressure

**Figure 5:**
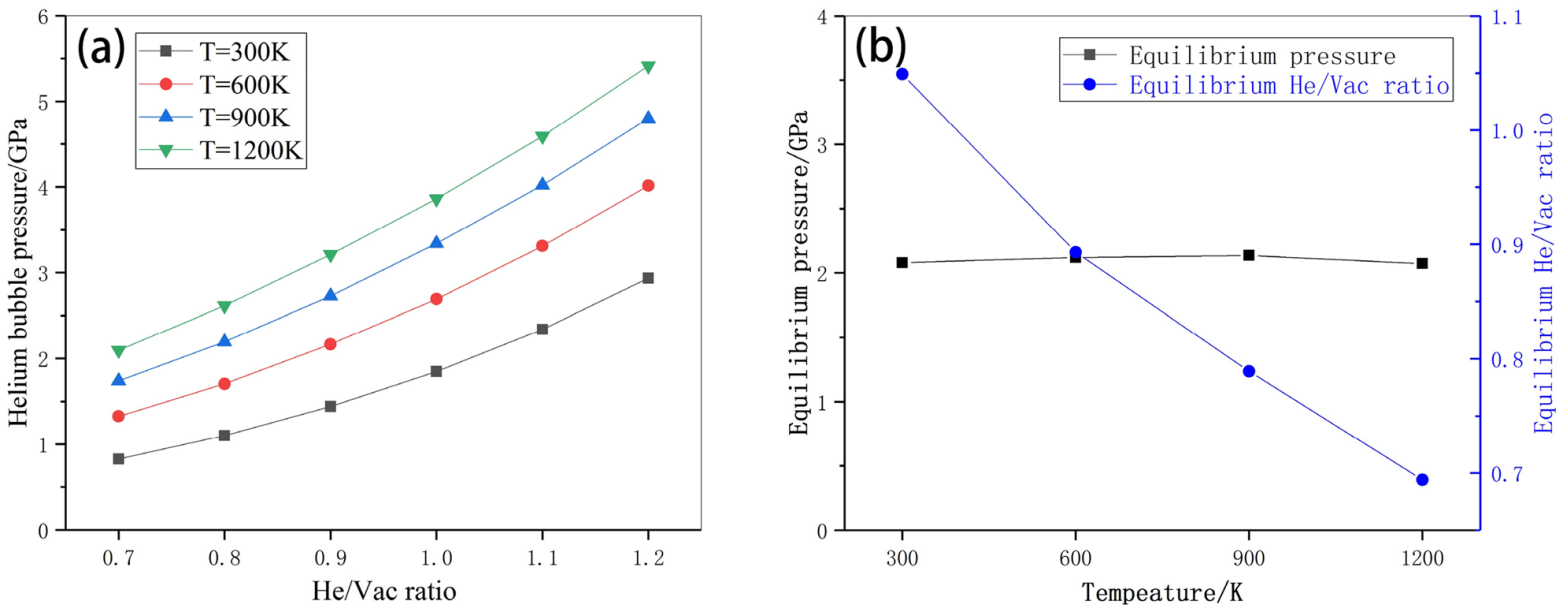
(**a**) Helium bubble pressure with respect to He/Vac ratio regarding four different temperatures. (**b**) Equilibrium pressure and equilibrium He/Vac ratio with respect to temperature.

A helium bubble with a diameter of 3 nm is taken as an example to study the effect of temperature on equilibrium pressure. Figure 5a,b show the variation of helium bubble pressure with respect to He/Vac ratio and its dependence on temperatures, respectively. It can be seen that: (1) the internal pressure increase with the He/Vac ratio and the temperature as shown in Fig. 5a. (2) The equilibrium He/Vac ratio decreases with the increase of temperature as given in Fig. 5b (in the blue line). This may be ascribed to the fact that high temperature implies the atoms in the helium bubble move or vibrate more rapidly, which results in the increase of internal pressure. Consequently, less helium atoms are required to balance the effects of surface energy. (3) Compared with the size effect, the effect of temperature on the equilibrium pressure is relatively weak as shown in Fig. 5b (in the black line), which decreases slightly with the increase of temperature. The reason for this phenomenon may be ascribed to two aspects. Firstly, the temperature influence on surface energy is quite small. For instance, the average surface energy is 3.299 J m^−2^ at 300 K, 3.278 J m^−2^ at 600 K, 3.267 J m^−2^ at 900 K and 3.224 J m^−2^ at 1200 K, respectively. Their relative difference is less than 3%. Secondly, no apparent change of the shape or configuration of the bubble is observed at the concerned temperature scale.

## Conclusions

In this paper, the effects of the bubble shape, bubble size and temperature on the equilibrium pressure are systematically investigated by means of molecular simulation. The major results are as follows:

1. An anomalous shape effect that the radial normal stress and mean stress distribution around a nanovoid or bubble are far from the spherical symmetry is found. For the case of nanovoid without internal pressure, its surrounding matrix is separated into 8 expansion regions and 6 contraction regions. This phenomenon is ascribed to geometry characteristic of polyhedron of the nanosized bubble and physical mechanism transition from crystal surfaces dominated to the ledges and triple junctions dominated.
2. A criterion is proposed to obtain the equilibrium pressure and equilibrium He/Vac ratio of helium bubbles, based on the evaluation of the average radial normal stress in spherical shells. This criterion is helpful to polish the basic concept of equilibrium pressure of bubbles, since no rigours definition of equilibrium pressure is provided or at least the traditional macroscopic definition of equilibrium pressure is not suitable for the case of nanoscale polyhedral bubbles. It is found that the equilibrium pressure increases with the decrease of helium bubble size, while temperature has little effect on the equilibrium pressure. Young–Laplace empirical formula overestimates the equilibrium pressure of helium bubbles in the tungsten.

The results obtained in this research are fundamental for understanding the mechanical state of helium-irradiated tungsten. With the shape effect in mind, consequent work may include the mechanisms of bubble coalesce, growth, interaction, radiation embrittlement and the equivalent surface energy of different materials at nanoscale.
